# Effects of local delivery of BMP2, zoledronate and their combination on bone microarchitecture, biomechanics and bone turnover in osteoporotic rabbits

**DOI:** 10.1038/srep28537

**Published:** 2016-06-22

**Authors:** Da Jing, Xuguang Hao, Fang Xu, Jian Liu, Fei Xu, Erping Luo, Guolin Meng

**Affiliations:** 1 Department of Biomedical Engineering, Fourth Military Medical University, Xi’an, China; 2 Institute of Orthopaedics, Xijing hospital, Fourth Military Medical University, Xi’an, China; 3 Department of Pharmacy, Zhejiang University of Technology, Hangzhou, Zhejiang, China; 4 Department of Radiation Oncology, PLA 302 hospital, Beijing, China; 5 Department of orthopaedics, the Fifth Hospital of Harbin, Harbin, China

## Abstract

The hip fracture is one major clinical challenge associated with osteoporosis, resulting in heavy socioeconomic burdens and high mortality. Systemic therapies of anti-osteoporosis drugs are expensive, time-consuming and also evoke substantial side effects, which fails to provide early protection from fractures. Accumulating evidence demonstrates the high bioavailability and therapeutic efficacy of local drug delivery in accelerating facture healing and bone defect repair. This study aims at investigating the effects of local delivery of BMP2 and zoledronate (two promising anabolic/anti-catobolic reagents) encapsulated by fibrin sealants into femoral necks on regulating bone quality and remodeling in osteoporotic rabbits subjected to combined ovariectomy and glucocorticoid injection. We show that 6-week BMP2 delivery exhibited more prominent effect on mitigating trabecular bone microarchitecture deterioration and mechanical strength reduction of femoral necks than local zoledronate treatment. BMP2 plus zoledronate showed more significant improvement of bone microstructure, mechanical strength and bone formation rate at 12 weeks post injection than single BMP2 or zoledronate delivery via μCT, biomechanical, histomorphometric and serum biochemical analyses. This study enriches our knowledge for understanding the availability of local drug delivery for improving bone quantity and quality, which may lead to earlier, safer and more efficient protection from osteoporosis-induced fractures in clinics.

Osteoporosis, a highly prevalent ‘progressive silent bone disease’ caused by age, disuse or disease, is characterized by notable bone mass loss and bone microarchitecture deterioration. Over 200 million people worldwide is afflicted by osteoporosis, and the number is expected to double by 2040^1^. Osteoporotic patients pose high risk of bone fractures, resulting in prominently increased disability, mortality, economic costs and social burdens. The femoral necks and vertebral bodies are the most common sites of fragility fractures, and the fracture patients have to receive long-term bed rest and are frequently complicated with bedsore, venous thrombosis of the lower extremity, and pulmonary and urinary system infection^2^. Hip fractures due to osteoporosis account for majority of medical expenditure and also lead to destructive impact on life quality and high mortality of patients (740 thousand deaths per year)^3^,^4^,^5^. Thus, exploring effective approaches for improving bone microarchitecture and fracture toughness in highly incident regions of fractures (*e.g.*, hips and vertebrae) holds great clinical and socioeconomic significance.

Current medications available for the prevention and treatment of osteoporosis mainly include calcium and vitamin D supplementation, bisphosphonates, calcitonin, parathyroid hormone and growth factors. Bone morphogenetic protein 2 (BMP2) and zoledronate are two of most promising anabolic and anti-catobolic reagents in clinics, respectively. BMPs are members of the transforming growth factor-β gene superfamily, which have exhibited notably stimulating effects on fracture healing, critical-sized bone defect repair and osteoporosis both experimentally and clinically via their strong anabolic actions^6^,^7^,^8^. BMP2 has the capacity of initiating a cascade of intracellular biochemical responses, resulting in the recruitment and differentiation of mesenchymal progenitor cells into osteoblasts^8^,^9^. BMP2 can also promote the biological functions of osteoblasts, and consequently accelerate the formation of bone matrix^10^,^11^. Zoledronate (ZOL) is a bisphosphonate that generally receives the once-yearly dosing regimen for the clinical treatment of osteoporosis via reducing bone turnover^12^,^13^. ZOL exerts its anti-catabolic effect by inactivating osteoclastic functions and promoting osteoclastic apoptosis^14^. However, systemic application of all above anti-osteoporosis drugs aims at enhancing whole-body bone mineral density (BMD). The use of systemic therapies is time-consuming and not able to provide early protection from fractures, and consequently fails to ensure high efficiency and high quality for improving local bone mass and bone strength at the sites with a high fracture incidence^15^. Moreover, systemic application of anti-osteoporosis drugs imposes heavier financial burdens for patients and also evokes substantial side effects, such as fever, pains, ulcers, gastrointestinal symptoms, and increases risk of cardiovascular diseases^16^,^17^,^18^.

Growing evidence has shown the high bioavailability and therapeutic efficacy of local drug delivery in bone diseases, which allows to directly focus the treatment on the targeted regions. It has been shown that local application of BMP2 significantly promotes spinal fusion, accelerates facture healing and enhances the repair of bone defects^19^,^20^,^21^. Studies have also demonstrated that local BMP2 delivery induced prominent increases of bone formation rate in osteoporotic animals^22^,^23^. Several investigations have revealed the high efficiency of local ZOL delivery for enhancing *in vivo* bone implant osteointegration and implant fixation via its prominent anti-catobolic effect^24^,^25^. Moreover, several studies have also shown the capacity of ZOL to promote the efficiency of BMP2 in repairing bone defects via composite scaffolds^26^,^27^. In spite of these positive findings, little is known about the effects of local delivery of BMP2, ZOL and their combination on bone remodeling and bone quality in highly incident fracture regions of osteoporotic animals.

In the present investigation, the osteoporotic rabbit model was established via the combination with ovariectomy and glucocorticoid injection. Then, the efficiency of local delivery into the femoral necks of BMP2, ZOL and BMP2 plus ZOL on bone loss were evaluated via systematic analysis for serum biochemical, bone biomechanical, μCT and histomorphometric parameters in osteoporotic rabbits. Our present study represents the first effort exploring the potential impacts of local application of BMP2, ZOL and their combination on bone microstructure, mechanical strength and bone turnover at the sites with high fracture incidence, and thus aims at improving local bone quantity and bone quality for minimizing the occurrence of osteoporotic fractures.

## Materials and Methods

### Animal model and experimental design

Thirty 5-month-old female New Zealand rabbits (2.50 ± 0.23 kg) were used in this experiment, which were obtained from the Animal Center of the Fourth Military Medical University, Xi’an, China). All animals were housed under controlled standard ambient temperature (23 ± 1 °C), relative humidity (50%~60%) and 12:12 h light-dark cycle (light on from 7 a.m. to 7 p.m.). All rabbits were allowed ad libitum access to clean tap water and standard laboratory chow (Animal Center of the Fourth Military Medical University, Xi’an, China) throughout the experimental period. Rabbits were acclimatized to the laboratory for 7 days prior to the surgery. The procedures in the experiment were approved by the Institutional Animal Care and Use Committee of the Fourth Military Medical University. All procedures were strictly carried out in accordance with the approved guidelines, and all efforts were made to minimize the number of animals used and their suffering.

**Figure 1:**
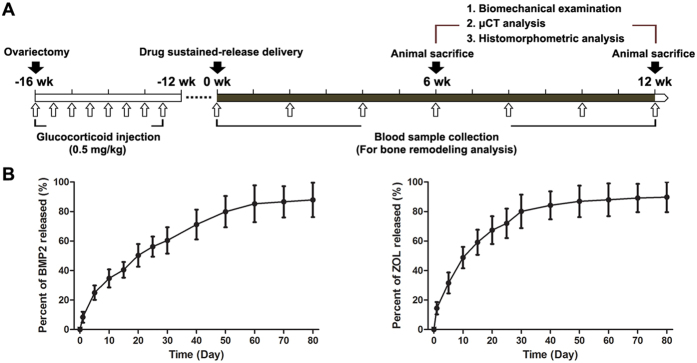
The experimental protocols for the present whole *in vivo* investigation, and the *in vitro* quantification of BMP2 and ZOL release kinetics. **(A)** The experimental protocols for the *in vivo* investigation. Rabbits were subjected to ovariectomy followed by intraperitoneal dexamethasone injection (a synthetic glucocorticoid) with 0.5 mg/kg twice per week for consecutive 4 weeks. Significant bone loss was confirmed at 16 weeks post surgery via dual energy X-ray absorptiometry examination. The sustained-release drugs were injected into the fundus of bilateral femoral necks of animals in the BMP2, ZOL, and BMP2 + ZOL groups. Blood samples were obtained from the rabbit marginal ear vein at 0, 2, 4, 6, 8, 10 and 12 weeks post drug injection for serum biochemical analysis. Three rabbits in each group were euthanatized after drug sustained-release delivery administration for 6 and 12 weeks. Left femora were harvested, wrapped in saline-soaked gauze and stored at −70 °C, which were used for mechanical testing. Right femora were harvested and immersed in 80% ethanol for μCT and histomorphometric analyses. **(B)** The *in vitro* quantification of BMP2 and ZOL release kinetics from the fibrin sealant (*n* = 4 for both BMP2 and ZOL quantification at each time point).

The experimental protocol of the present study is shown in Fig. 1A. Rabbits were divided into five equal groups (*n* = 6) and randomly assigned to the sham-operated control group (Control), ovariectomy combined with glucocorticoid injection group (OVX + GLU), OVX + GLU with BMP2 injection group (BMP2), OVX + GLU with zoledronate injection group (ZOL), and OVX + GLU with combined injection of BMP2 and zoledronate group (BMP2 + ZOL). Before subjected to ovariectomized or sham-control surgery, animals were anesthetized via intramuscular injection of sumianxin II (0.2 ml/kg) and pentobarbital sodium (3%, 1 ml/kg). The dorsum of the rabbit was shaved, cleansed with iodophor solution, and then covered with sterile drapes. A single longitudinal skin incision was made on the dorsal midline at the level of the kidneys. Both ovaries of rabbits in the OVX + GLU, BMP2, ZOL and BMP2 + ZOL groups were ligated and removed. Success of ovariectomy was confirmed at necropsy by failure to detect ovarian tissue and by observation of marked atrophy of uterine horns. The rabbits in the sham group underwent sham surgery, during which time the ovaries were exposed and kept intact. To avoid potential wound infection, each animal was injected with penicillin (40000 U) per day via intramuscular injection for 3 consecutive days post surgery. The OVX animals were subjected to intraperitoneal dexamethasone injection (a synthetic glucocorticoid) with 0.5 mg/kg twice per week for consecutive 4 weeks. The BMD values of lumbar vertebra were examined by using a dual energy X-ray absorptiometry system (DPX-IQ, GE healthcare, Madison, WI) at 1 day pre-surgery and 1, 2 and 4 months post surgery, respectively. As shown in Table 1, the OVX rabbits exhibited significant decreases in vertebral BMD as compared with the sham-operated rabbits since 2 months post surgery. The rabbit at 4 months post surgery with the vertebral BMD higher than the mean value minus two standard deviation of pre-surgery BMD of all animals was regarded as qualified osteoporotic animal^28^.

### Establishment of the drug sustained-release delivery system

Lyophilized human fibrinogen (100 mg, Shanghai RAAS Blood Products Co, Ltd, Shanghai, China) was dissolved in 4 ml sterile water for injection to a final concentration of 25 mg/ml. This human fibrinogen solution (0.5 ml) was mixed with 1 ml recombined human BMP2 solution (0.2 mg/ml in sterile saline), 1 ml zoledronate solution (0.1 mg/ml in sterile saline, Zometa, Novartis Pharmaceuticals East Hanover, NJ), or 0.5 ml BMP2 plus 0.5 ml zoledronate solution. The mixture was then extracted with a syringe. Lyophilized human thrombin (1000 IU, Shanghai RAAS Blood Products Co, Ltd) was dissolved in 4 ml, 40 mM CaCl_2_ solution. Then, the thrombin solution with 0.5 ml was extracted with another syringe. The two syringes were mounted on a duplex syringe supporter. The two syringes were interconnected with a Y-shape connecting pipe, and a needle was installed at the end of the Y-shape connecting pipe, which could be used for sustained-release delivery of drugs into the femoral necks of rabbits.

For local drug sustained-release delivery, the rabbits with confirmed osteopenia after OVX and glucocorticoid injection were anesthetized via intramuscular injection with pentobarbital sodium (3%, 30 mg/kg). Bilateral hindlimbs of animals were shaved, cleansed with iodophor solution, and covered with sterile drapes. The needle of the Y-shape connecting pipe was inserted at the site 1 cm beneath the femoral trochanter with 1.5 cm needling depth. The sustained-release drugs were slowly injected into the fundus of bilateral femoral necks. Blood samples were obtained from the rabbit marginal ear vein at 0, 2, 4, 6, 8, 10 and 12 weeks post drug injection. Serum samples were obtained by centrifuging the blood samples for 20 min and stored at −70 °C for biochemical analysis. Three rabbits in each group were euthanatized with an overdose of pentobarbital sodium (Sigma, St. Louis, MO) after the drug sustained-release delivery for 6 and 12 weeks. All rabbits received intramuscular injections of 50 mg/kg tetracycline at 14 and 13 days and 8 mg/kg calcein at 4 and 3 days before sacrifice, respectively. Left femora were harvested, wrapped in saline-soaked gauze and stored at −70 °C, which were used for mechanical testing. Right femora were harvested and immersed in 80% ethanol for μCT and histomorphometric analyses.

For *in vitro* analysis of BMP2 and ZOL release kinetics, the sustained-release drugs (BMP2 and ZOL encapsulated by the fibrin sealants) were dispersed in the PBS solution (PH: 7.4). The mixture was placed in a shaking incubator with 37 °C environment. The supernatant was extracted at 1, 5, 10, 15, 20, 25, 30, 40, 50, 60, 70 and 80 days post incubation. After each supernatant extraction, new PBS solution (PH: 7.4) with equal volume was immediately supplemented. The BMP2 concentrations at each time point were quantified by using the commercial ELISA kits (Wuhan Boster Biotech co. Ltd., Wuhan, China) according the protocol provided by the manufacturers. The ZOL released levels were analyzed by using the high performance liquid chromatography (Agilent 1100, Agilent Technologies, Santa Clara, CA). The *in vitro* quantification of BMP2 and ZOL release kinetics are shown in Fig. 1B.

### Serum biochemical analysis

Serum ALP (a biological marker of bone formation) and serum TRACP 5b (a bone resorption marker) in the rabbits of the five groups were quantified with commercial ELISA kits (Beyotime, Shanghai, China). The measurements of serum ALP and TRACP 5b concentrations of rabbits were performed according to the protocols provided by the manufacturers.

### Biomechanical examination

After thawing at room temperature for 1 h, the necks of left femurs were used for mechanical shear testing via a biomechanical testing system (MTS 858 Mini Bionix II, MTS Systems Corp., Eden Prairie, MN). The proximal half of the left femoral sample was fixed in a drill chuck for mechanical shear test of the femur neck. Load was applied at a constant displacement rate of 0.2 mm/min until fracture occurred. The inner and outer width and height of the femur at the point of fracture were measured with a vernier caliper. The force and displacement data were automatically recorded into a computer which was interfaced to the material testing machine and the load-displacement curve was plotted simultaneously. The stress-strain curve was plotted based on the load-displacement curve, in which force and displacement were normalized to be stress and strain by the dimensions of bone samples. The following indices were directly determined from the stress-strain curve, including shear stress, shear strain and shear modulus.

### μCT analyses

A high-resolution μCT machine (GE healthcare, Madison, WI) was used to evaluate skeletal microarchitecture in the right femurs (*n* = 6 for each group). The basic parameters of the scanner were the following: voltage 80 kV, current 80 μA, exposure time 2.96 s, total rotation angle 360^o^ and rotation angel of increment 0.5^o^. The scanning resolution was 14 μm/slice. The femoral samples were placed in a 20-mm-diameter sample tube perpendicularly to the scanning axis with a total reconstructed height of 12 mm. One scan took about 30 min. After scanning, the image data were transferred to a workstation and 3-D reconstruction was performed for visualization and data analysis. A cylindrical volume of interest (VOI) with 3.0-mm diameter and 3.0-mm height was selected for the analysis of trabecular bone microarchitecture in the femoral neck. The trabecular bone microarchitecture could be visually and quantitatively analyzed with the MicroView program (GE healthcare, Madison, WI). The quantitative analysis of the trabecular bone microstructure included the following indices, trabecular bone mineral content (BMC), trabecular BMD (BMD), trabecular number (Tb.N), trabecular separation (Tb.Sp), trabecular thickness (Tb.Th), bone volume with total volume as referent (BV/TV), bone surface with bone volume as referent (BS/BV), and connectivity density (Conn.D).

### Bone histomorphometry

After μCT scanning, samples were embedded in methylmethacrylate. The LEICA 2500E diamond saw microtome (Leica SpA, Milan, Italy) was used to section the femoral intertrochanteric cross with the longitudinally (~60 μm thick). These undecalcified sections with tetracycline and calcein double staining were imaged using a fluorescence microscope (LEICA DM LA, Leica Microsystems, Heidelberg, Germany) to quantify the dynamic trabecular bone histomorphometric parameters (*n* = 6 for each group), including mineral apposition rate (MAR), mineralizing surface per bone surface (MS/BS) and bone formation rate per bone surface (BFR/BS). For histomorphometric analysis, one section was measured for each sample, and all sections were coded and analyzed ‘blind’ by one independent observer. The VOI used for the histomorphometric analysis kept consistent with the VOI selected in the μCT analysis. Two repeated measurements were performed for each section within the selected VOI.

### Statistical analysis

Statistical analyses were performed using SPSS version 13.0 for Windows software (SPSS, Chicago, IL). All data presented in this study were examined for normal distribution using the Kolmogorov-Smirnov test, and evaluated for homogeneity of variance using the Levene’s test. Analyses showed that each specific parameter in the three groups obeyed normal distribution and homoscedasticity. One-way analysis of variance (ANOVA) was employed for evaluating the existence of differences among the five groups and once a significant difference was detected, Fisher’s least significant difference (LSD) t-test was used to determine the significance between every two groups. The significance level was set at 0.05.

## Results

### Serum biochemical examination

**Figure 2:**
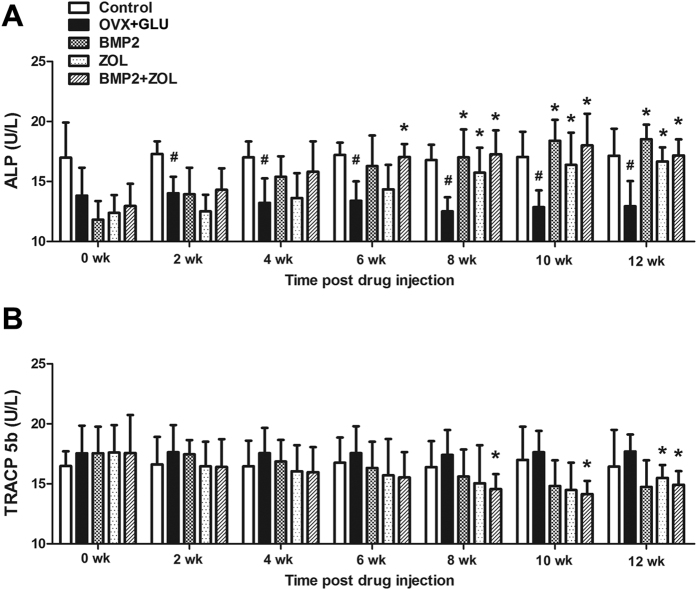
Effects of local drug sustained-release delivery (including BMP2, zoledronate and their combination) into bilateral femoral necks on serum biochemical indices (bone turnover markers) in rabbits subjected to ovariectomy combined with glucocorticoid injection (*n* = 6 in each group at 0, 2, 4 and 6 weeks post drug sustained-release delivery, and *n* = 3 at 8, 10 and 12 weeks post drug delivery), including bone formation markers **(A)** alkaline phosphatase (ALP) and bone resorption markers **(B)** tartrate-resistant acid phosphatase 5b (TRACP 5b). Control, the control group; OVX + GLU, ovariectomy combined with glucocorticoid injection group; BMP2, OVX + GLU with BMP2 injection group; ZOL, OVX + GLU with zoledronate injection group; BMP2 + ZOL, OVX + GLU with combined injection of BMP2 and zoledronate group. Values are all expressed as mean ± S.D. ^#^Significant difference from the Control group with *P* < 0.05; ^*^Significant difference from the OVX + GLU group with *P* < 0.05.

As shown in Fig. 2A, serum ALP levels in the OVX + GLU group were significantly lower than those in the Control group at 2, 4, 6, 8, 10 and 12 weeks post drug sustained-release delivery (*P* < 0.05). The BMP2 group and ZOL group exhibited significantly higher serum ALP concentrations than the OVX + GLU group at 8, 10 and 12 weeks post injection of sustained-release drugs (*P* < 0.05). Furthermore, serum ALP levels in the BMP2 + ZOL group were significantly higher than those in the OVX + GLU group at 6, 8, 10 and 12 weeks post local drug delivery (*P* < 0.05). As shown in Fig. 2B, the OVX + GLU group did not exhibit statistically significant difference in serum TRACP 5b concentrations as compared with the Control group at 0, 2, 4, 6, 8, 10 or 12 weeks post drug injection. No significant difference was found in serum TRACP 5b concentrations between the OVX + GLU and BMP2 groups throughout the experimental period. The ZOL group only exhibited significantly lower serum TRACP 5b levels than the OVX + GLU group at 12 weeks post local drug sustained-release delivery (*P* < 0.05). Moreover, serum TRACP 5b levels in the OVX + GLU group was significantly lower than those in the OVX + GLU group at 8, 10 and 12 weeks post injection of sustained-release drugs (*P* < 0.05).

### Biomechanical testing

**Figure 3:**
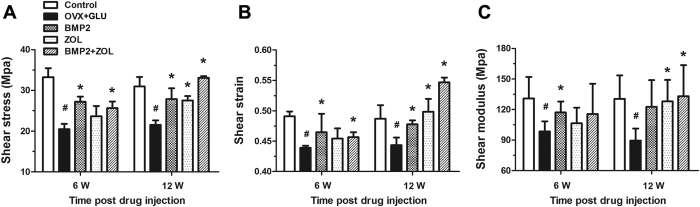
Effects of local drug sustained-release delivery (including BMP2, zoledronate and their combination) into bilateral femoral necks on femoral biomechanical properties in rabbits subjected to ovariectomy combined with glucocorticoid injection, including **(A)** shear stress, **(B)** shear strain, and **(C)** shear modulus. Control, the control group; OVX + GLU, ovariectomy combined with glucocorticoid injection group; BMP2, OVX + GLU with BMP2 injection group; ZOL, OVX + GLU with zoledronate injection group; BMP2 + ZOL, OVX + GLU with combined injection of BMP2 and zoledronate group. Values are all expressed as mean ± S.D. ^#^Significant difference from the Control group with *P* < 0.05; ^*^Significant difference from the OVX + GLU group with *P* < 0.05.

The results of the mechanical properties of femoral necks via mechanical shear testing are shown in Fig. 3. The OVX + GLU rabbits showed significant decreases in shear stress, shear strain and shear modulus in comparison with the Control rabbits at both 6 and 12 weeks post drug sustained-release delivery (*P* < 0.05). Rabbits in the BMP2 group and BMP2 + ZOL group exhibited significant enhancement in shear stress and shear strain as compared with the OVX + GLU rabbits at both 6 and 12 weeks post local sustained-release drug delivery (*P* < 0.05). The BMP2 group also showed significantly higher shear modulus than the OVX + GLU group at 6 weeks post injection (*P* < 0.05). Moreover, the ZOL group and BMP2 + ZOL group displayed significantly higher shear modulus levels than the OVX + GLU group at 12 weeks post local drug delivery (*P* < 0.05).

### μCT analysis

**Figure 4:**
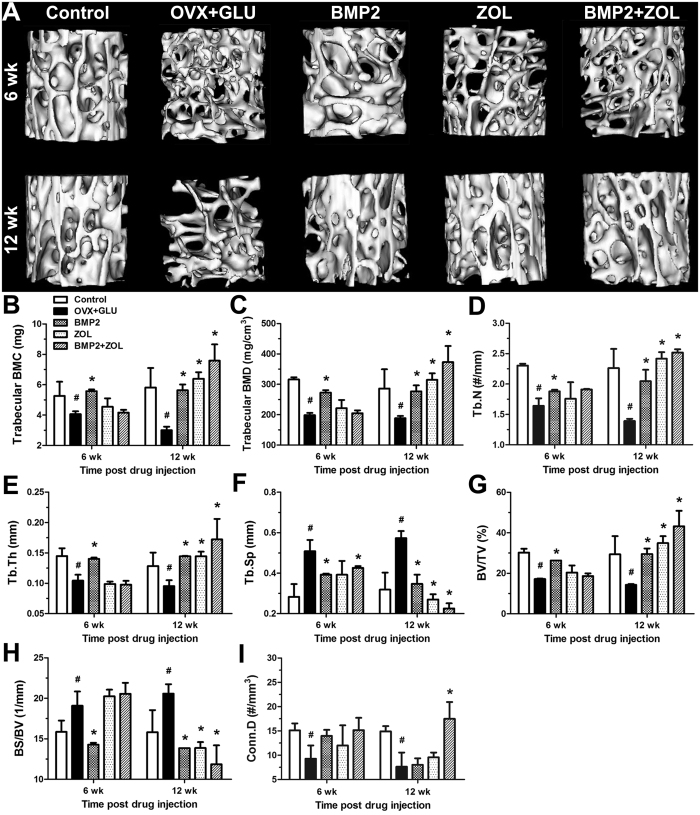
Effects of local drug sustained-release delivery (including BMP2, zoledronate and their combination) into bilateral femoral necks on trabecular bone microarchitecture in rabbits subjected to ovariectomy combined with glucocorticoid injection. (**A**) 3-D MicroCT images of trabecular bone microarchitecture determined by the cylindric VOI. (**B~I**) Statistical comparisons of indices of trabecular bone microarchitecture, including **(B)** trabecular bone mineral content (BMC), **(C)** trabecular bone mineral density (BMD), **(D)** trabecular number (Tb.N), **(E)** trabecular thickness (Tb.Th), **(F)** trabecular separation (Tb.Sp), **(G)** bone volume per tissue volume (BV/TV), **(H)** bone surface per bone volume (BS/BV), and **(I)** connectivity density (Conn.D). Control, the control group; OVX + GLU, ovariectomy combined with glucocorticoid injection group; BMP2, OVX + GLU with BMP2 injection group; ZOL, OVX + GLU with zoledronate injection group; BMP2 + ZOL, OVX + GLU with combined injection of BMP2 and zoledronate group. Values are all expressed as mean ± S.D. ^#^Significant difference from the Control group with *P* < 0.05; ^*^Significant difference from the OVX + GLU group with *P* < 0.05.

Representative μCT images for trabecular bone microarchitecture in the five experimental groups are shown in Fig. 4A. The femoral necks from the OVX + GLU rabbits displayed notable reduction in the trabecular number, trabecular connection, trabecular area and cortical thickness as compared with those in the Control group. Local BMP2 devliery for 6 weeks exhibited obvious enhancement in trabecular number and thickness in the osteoporotic rabbits. Significant improvement in cancellous bone microarchitecture was observed in the BMP2, ZOL and BMP2 + ZOL groups at 12 weeks post drug delivery in comparision with the OVX + GLU group, especially prominent in the BMP2 + ZOL group. Statistical comparisons (Fig. 4B~I) demonstrate that ovariectomy combined with glucocorticoid injection induced significant decreases of trabecular BMC, trabecular BMD, Tb.N, Tb.Th, BV/TV and Conn.D, and increases of Tb.Sp and BS/BV at 6 weeks and 12 weeks post drug sustained-release delivery (*P* < 0.05). The BMP2 group showed significantly higher trabecular BMC, trabecular BMD, Tb.N, Tb.Th and BV/TV, and lower Tb.Sp and BS/BV than the OVX + GLU group at 6 and 12 weeks post injection (*P* < 0.05). No significant improvement for local ZOL delivery in any trabecular bone parameter as compared with the OVX + GLU group at 6 weeks post injection. The ZOL group showed higher trabecular BMC, trabecular BMD, Tb.N, Tb.Th and BV/TV, and lower Tb.Sp and BS/BV than the OVX + GLU group at 12 weeks post injection of sustained-release drugs (*P* < 0.05). Furthermore, the BMP2 + ZOL group exhibited significant increase in Tb.N and decrease in Tb.Sp than the OVX + GLU group at 6 weeks post sustained-release drug injection (*P* < 0.05). The BMP2 + ZOL group also showed significantly higher trabecular BMC, trabecular BMD, Tb.N, Tb.Th, BV/TV and Conn.D levels, and lower Tb.Sp and BS/BV levels at 12 weeks post drug injection (*P* < 0.01).

### Bone histomorphometry

**Figure 5:**
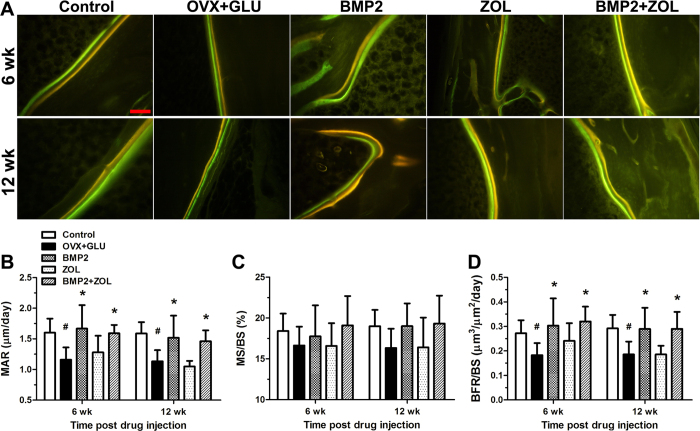
Effects of local drug sustained-release delivery (including BMP2, zoledronate and their combination) into bilateral femoral necks on trabecular bone histomorphometry in rabbits subjected to ovariectomy combined with glucocorticoid injection. (**A**) Representative tetracycline and calcein double-labeling sections in the regions of femoral necks. Scale bar represents 100 μm for all images. (**B~D**) Comparisons of dynamic histomorphometric parameters of trabecular bone by double labeling with tetracycline and calcein, including (**B**) mineral apposition rate (MAR), (**C**) mineralizing surface per bone surface (MS/BS) and (**D**) bone formation rate per bone surface (BFR/BS). Control, the control group; OVX + GLU, ovariectomy combined with glucocorticoid injection group; BMP2, OVX + GLU with BMP2 injection group; ZOL, OVX + GLU with zoledronate injection group; BMP2 + ZOL, OVX + GLU with combined injection of BMP2 and zoledronate group. Values are all expressed as mean ± S.D. ^#^Significant difference from the Control group with *P* < 0.05; ^*^Significant difference from the OVX + GLU group with *P* < 0.05.

Representative images of dynamic histomorphometric analysis in the five groups via tetracycline and calcein dual labeling are shown in Fig. 5A. The OVX + GLU rabbits exhibit lower cancellous bone mineralization rate than the Control group at 6 and 12 weeks post drug injection. Obvious increase of mineralization rate was found in the BMP2 and BMP2 + ZOL groups at 6 and 12 weeks post local drug delivery as compared with the OVX + GLU group. Furthermore, quantitative comparisons of the dynamic histomorphometric parameters (Fig. 5B~D) demonstrate that the OVX + GLU rabbits showed significantly lower MAR and BFR/BS than the Control group at 6 and 12 weeks post drug injection (*P* < 0.05). In comparison with the OVX + GLU group, rabbits in the BMP2 and BMP2 + ZOL groups exhibited significantly increased MAR and BFR/BS at 6 and 12 weeks post injection of sustained-release drugs (*P* < 0.05). However, rabbits in the ZOL group did not show obvious increase in MAR or BFR/BS as compared with the OVX + GLU group. In addition, no significant difference in the MS/BS levels was observed among the five groups.

## Discussion

Enhancement of bone mass and mechanical strength in highly incident regions of fractures (*e.g.*, hips and vertebrae) is of great clinical significance of lowering osteoporosis-related morbidity and mortality rate. In view of the limitation of systemic therapies, several investigations have reported the high efficiency of local delivery of osteogenic agents in repairing bone fractures and enhancing implant osseointegration^15^,^29^,^30^, whereas the potential effects of local drug delivery on improving local bone quantity and quality at the sites with high fracture incidence of osteoporotic skeletons remain poorly understood. In the present study, we systematically evaluated the impacts of local delivery into the femoral necks with BMP2, ZOL and BMP2 plus ZOL on bone microarchitecture, bone quality and bone remodeling in osteoporotic rabbits. Our findings reveal that local BMP2 delivery for 6 weeks exhibited prominent effect on mitigating the decrease of bone loss, and BMP2 plus ZOL showed most significant improvement of bone mass and bone strength at 12 weeks post injection. This study enriches our basic knowledge for understanding the availability of local drug delivery for improving local bone quality, which may provide more efficient and high-quality protection from fractures for osteoporotic patients in clinics.

Our present study employed fibrin sealants as the carrier for the local delivery and controlled release of osteogenic reagents. Fibrin sealants, derived from plasma coagulation proteins, have been widely used for promoting hemostasis, wound healing and defect closure since the 1980s due to its nontoxicity and excellent biocompatiblity and biodegradability^31^. Fibrin sealants have two components, fibrinogen and thrombin. Fibrinogen, as the main structural component in fibrin sealants, is activated by thrombin and then converted to a three dimensional spongy fibrin polymer with high similarity to a physiological clot, resembling the last stage of a natural physiological coagulation process. Several previous investigations demonstrate that the mechanical tensile strength of fibrin sealants was directly related with the fibrinogen concentration^32^,^33^. Our present study employed fibrin sealants with 25 mg/mL fibrinogen, which exhibited approximately 40 kPa tensile strength according to the experimental results by Alston *et al*^33^. Recent studies have also reported the application of fibrin sealants as an effective vehicle for the delivery of cells, drugs or growth factors^34^,^35^. Drugs loaded into the fibrin sealants can be encapsulated by the fibrin gel, resulting in controlled drug sustained-release with the accompanying resorption and degradation of fibrin sealants. Several investigations have also revealed the ability of fibrin sealants with favorable biocompatiblity with bone tissues and excellent osteogenic potential^36^,^37^. Therefore, fibrin sealants might have the potential to be employed as an efficient carrier for sustained release of anti-osteoporosis drugs.

In the present study, combined ovariectomy and glucocorticoid injection were employed to induce osteopenia in rabbits, which exhibited significant reduction of bone formation, as evidenced by decreased serum ALP concentrations and cancellous bone MAR and BFR/BS. These results keep consistent with several previous findings by Schorlemmer *et al*.^38^. BMPs are well known as efficient osteoinductive molecules with the ability to stimulate bone formation by inducing the recruitment and differentiation of bone marrow stromal cells^8^,^9^. However, BMPs can rapidly lose their bioactivity when they are subjected to the systemic administration^39^,^40^, and thus a delivery carrier of BMPs with sustained drug release exhibits superiority to the systemic burst release. We herein demonstrate that the circulating ALP concentrations were obviously elevated since 4 weeks of local injection with BMP2. Local delivery BMP2 also maintained the trabecular MAR and BFR/BS in significantly higher levels in femoral necks of osteoporotic rabbits at 6 weeks and up to 12 weeks post drug injection. Thus, our findings demonstrate that decreased bone formation in osteoporotic animals is able to be promoted rapidly by local BMP2 sustained-release delivery, as well as be maintained in long-term constantly higher levels. ZOL is currently the most prevalent drug in the bisphosphonates family with its prominent antiresorptive effect on osteoporosis via intravenous injection, whereas the side effects and low bioavailability remain unnegligible clinical issues. In the present study, the femoral neck is the only part of the skeleton directly exposed to ZOL. Our findings show that local ZOL delivery exhibited no significant improvement in trabecular MAR or BFR/BS throughout the experimental period, and only increased the serum TRACP 5b levels at 12 weeks post injection. Thus, our results reveal that local BMP2 injection exerts more rapid and efficient promotional effect on bone formation than the antiresorptive effect of local ZOL injection, which may contribute to differential time-dependent effect of both agents and their combination on regulating bone microarchitecture and bone quality. Moreover, it has been previously shown that serum sclerostin levels exhibit sharply rapid increase and reach the peak within one week of ZOL injection^41^. Since sclerostin is a critical negative regulator for osteoblastogenesis-associated Wnt/β-catenin signaling and BMP signaling, the sharply rapid increase of serum sclerostin levels after ZOL injection may be also one possible reason why the BMP2 + ZOL group exhibited no significant improvement than single BMP2 group on bone quantity and bone quality at 6 weeks post drug injection.

Impaired cancellous bone 3-D microstructure and decreased cancellous bone thickness are believed to be early events for the occurrence of osteoporosis^42^,^43^. In the present study, rabbits subjected to combined ovariectomy and glucocorticoid injection exhibited significant deterioration of trabecular bone microarchitecture in femoral necks, which kept consistent with previous findings^44^,^45^. Furthermore, our results reveal a time-dependent effect of local delivery of BMP2 and ZOL on trabecular bone microarchitecture of femoral necks. First, local BMP2 injection exhibits early protection from trabecular bone deterioration at 6 weeks post injection, as evidenced by increased trabecular BMC, BMD, Tb.N, Tb.Th, BV/TV and Conn.D, and decreased Tb.Sp and BS/BV. However, 12-week BMP2 injection did not show significant improvement in trabecular bone microarchitecture as compared with 6-week BMP2 injection. Second, we found that ZOL injection for 12 weeks, but not for 6 weeks, exhibited significantly inhibitive effect on bone mass loss and trabecular bone deterioration in rabbits subjected to ovariectomy and glucocorticoid injection. These results keep consistent with our findings in bone turnover, demonstrating that local ZOL delivery exerts its relatively lagging anti-catabolic effect than the anabolic action of BMP2 on inhibiting osteopenia. Third, the BMP2 + ZOL group at 6 weeks post drug injection did not show any improvement in trabecular bone microarchitecture as compared with the single drug injection. However, BMP2 plus ZOL injection for 12 weeks showed most prominent efficiency on preventing trabecular bone deterioration, revealing the synergistic additive effects of BMP2 and ZOL on the regulation of bone remodeling.

Patients with femoral neck fractures tend to experience more shear force, which is thus considered to be an excellent indicator for fracture toughness in the proximal femur^46^,^47^. It has been shown that the shear forces exhibit a high correlation with the BMD of the human proximal femur^48^. Our findings demonstrate that local BMP2 delivery from fibrin sealants exhibited more prominent effect on improving mechanical properties than the ZOL delivery at 6 weeks post drug injection. Moreover, we also show that BMP2 plus ZOL delivery for 12 weeks exhibited stronger efficiency on promoting bone structural and material properties than BMP2 or ZOL injection, as evidenced by significantly increased shear stress, shear strain and modulus. Our findings on mechanical properties in femoral necks keep consistent with the results of bone turnover and bone microstructure, and revealed the dependence of fracture resistance capacity on trabecular bone mass and bone microarchitecture. Our results demonstrate the difference of local BMP2 and ZOL application in regulating bone quality in rabbits with osteopenia, which have significant clinical implications for reducing the risks of osteoporotic fractures.

In the present study, we only used single-dose BMP2 and ZOL encapsulated by fibrin sealants as local sustained-release systems to investigate their osteoprotective effects. We observed the differential time-dependent effect of local BMP2, ZOL and BMP2 + ZOL delivery on bone microarchitecture, mechanical properties and bone turnover in highly incident fracture regions of osteoporotic rabbits. More importantly, our study also revealed the potential of fibrin sealants as an effective carrier for encapsulating anti-osteoporosis drugs (*e.g.*, BMP2 and zoledronate) for improving bone quantity and quality in highly incident fracture regions of osteoporotic animals. However, it should be noted that local delivery of BMP2 and ZOL with different concentrations may result in differential effects on regulating bone quantity, bone quality and bone turnover. Thus, this interesting topic will be systematically investigated via larger-scale animal experiments in our succeeding studies, which may help provide more valuable clues for more efficient application of local drug delivery based on fibrin sealants for protecting from osteoporosis-associated fractures.

In conclusion, our study represents the first published data demonstrating the differential time-dependent effect of local BMP2, ZOL and BMP2 + ZOL delivery on bone microarchitecture, mechanical properties and bone turnover in highly incident fracture regions of osteoporotic animals. We observed earlier protection of local BMP2 delivery from osteoporosis-induced decreases of bone quantity and bone quality than ZOL, and BMP2 plus ZOL exhibited most significant long-term efficiency on resisting bone loss and enhancing the fracture toughness. This study highlights the potential of local drug delivery for improving skeletal structural and mechanical properties at the sites with high fracture incidence, which may lead to earlier, safer and more efficient protection from osteoporosis-associated fractures in clinics.

## Additional Information

**How to cite this article**: Jing, D. *et al*. Effects of local delivery of BMP2, zoledronate and their combination on bone microarchitecture, biomechanics and bone turnover in osteoporotic rabbits. *Sci. Rep.* **6**, 28537; doi: 10.1038/srep28537 (2016).

## Figures and Tables

**Table 1:** The values of vertebral bone mineral density for the rabbits subjected to ovariectomy (OVX) or sham operation before surgery and at 1, 2 and 4 months post surgery.

**BMD (mg/cm**^**3**^)	**Pre-surgery**	**Post-surgery**		
		**1 month**	**2 months**	**4 months**
OVX animals (*n* = 24)	315.0 ± 30.7	301.3 ± 35.1	270.6 ± 36.5^*^	242.6 ± 36.6^*^
Sham-operated animals (*n* = 6)	323.5 ± 34.8	319.8 ± 37.6	324.9 ± 39.1	329.8 ± 38.6

Values are expressed as mean ± S.D. ^*^Significant difference from the sham-operated rabbits with *P* < 0.05.
